# Real-Time Non-Contact Millimeter Wave Radar-Based Vital Sign Detection

**DOI:** 10.3390/s22197560

**Published:** 2022-10-06

**Authors:** Zhiqiang Gao, Luqman Ali, Cong Wang, Ruizhi Liu, Chunwei Wang, Cheng Qian, Hokun Sung, Fanyi Meng

**Affiliations:** 1 School of Astronautics, Harbin Institute of Technology, Harbin 150001, China; 2 School of Information and Communication, Harbin Institute of Technology, Harbin 150001, China; 3 Ocean College, Zhejiang University, Hangzhou 310027, China; 4 Korea Advanced Nano Fab Center (KANC), Suwon-si 443270, Korea

**Keywords:** detection, Doppler frequency shift, heartbeat detection, Fourier transform, mm-wave radar

## Abstract

In this paper, the extraction of the life activity spectrum based on the millimeter (mm) wave radar is designed to realize the detection of target objects and the threshold trigger module. The maximum likelihood estimation method is selected to complete the design of the average early warning probability trigger function. The threshold trigger module is designed for the echo signal of static objects in the echo signal. It will interfere with the extraction of Doppler frequency shift results. The moving target detection method is selected, and the filter is designed. The static clutter interference is filtered without affecting the phase difference between the detection sequences, and the highlight target signal is improved. The frequency and displacement of thoracic movement are used as the detection data. Through the Fourier transform calculation of the sequence, the spectrum value is extracted within the estimated range of the heartbeat and respiration spectrum, and the heartbeat and respiration signals are picked up. The proposed design uses Modelsim and Quartus for CO-simulation to complete the simulation verification of the function, extract the number of logical units occupied by computing resources, and verify the algorithm with the vital signs experiment. The heartbeat and respiration were detected using the sports bracelet; the relative errors of heartbeat detection were 0–6.3%, the respiration detection was 0–9.5%, and the relative errors of heartbeat detection were overwhelmingly less than 5%.

## 1. Introduction

Over the past few decades, non-contact human vital sign monitoring received much attention [1,2,3,4,5,6]. The connection of electrodes or sensors to the human body is necessary for traditional heartbeat monitoring systems, such as electrocardiography (ECG) and photoplethysmography (PPG), to collect vital indicators, such as the rate of breathing or the heartbeat. It is not easy and always practicable, particularly for long-term monitoring applications or individuals with sensitive skin. Non-contact sensors that can track a person’s heartbeat would have many different uses. For instance, older people and kids were monitored as they slept [4], as well as patients, over an extended time at home [5,6].

The viability of utilizing a Doppler radar to monitor critical human indicators was examined by several research groups [4,5,6,7,8]. The chest wall will shift very little with each pulse and respiratory cycle, which the radar can detect. Chest wall displacement and respiration rates are encoded in the phase of the reflected signal from the chest. The majority of related research focuses on these methods and hardware design. Some researchers concentrated on the latter and made notable advancements in transceiver circuit development and improvement [9,10,11], as well as system architecture development and improvement [12,13,14]. Realizing mm-wave radar’s ability to detect critical human indicators in real time is crucial [6,7,8].

However, two serious problems exist with the baseband signal processing methods for non-contact vital sign identification. The limited resolution of the current frequency estimators is the first problem. Currently, approaches for heartbeat identification include small-angle approximation [5], modified arctangent demodulation [7], feature-based correlation [8], and frequency–time phase regression (FTPR) [15]. However, approaches that can offer better frequency resolutions are still required [3,16]. The difficulty comes from the fact that the chest wall motions caused by the pulse are quite modest and can, thus, be readily hidden by the higher harmonics of the respiration signal due to smearing, leaking, and windowing effects.

Instead of an electrical signal, as in the case of ECG sensors, Doppler radars detect the mechanical signal of the chest wall vibration. Researchers can identify the best methods to retrieve the needed information by understanding this mechanical signal’s temporal and frequency domain behaviors and using correct mathematical representation [15]. A sine wave is frequently used to mimic the mechanical signal of the human pulse [1,2,3,4,5,6,7,8]. Based on the field-programmable gate array (FPGA) hardware architecture, mm-wave radar can monitor human life activities and achieve non-contact between detecting equipment and the targeted item [17,18,19,20,21,22,23,24,25].

It is inferred from the above discussion that real-time non-contact mm-wave radar-based vital sign detection is essential for the heartbeat and respiration system. The proposed design verifies the algorithm with vital sign experimentation, and the relative error was observed to be very low (less than 5%) for heartbeat detection. The methodology of mm-wave radar for the proposed work is explained in Section 2, and the targeted frequency algorithm for this study is expressed in Section 3. The heartbeat and respiratory were detected using a sports bracelet defined in Section 4.

## 2. Analysis of mm-Wave Radar Detection Principles

**Figure 1:**
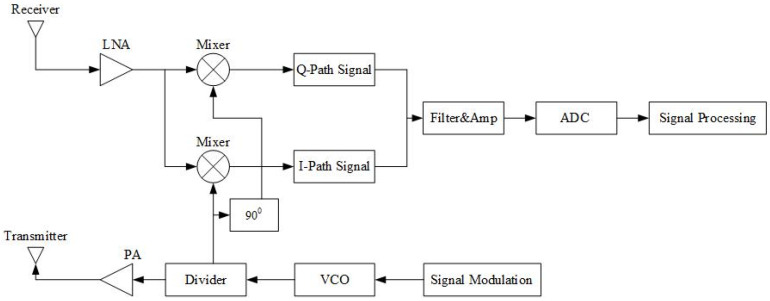
Linear frequency modulated continuous wave (LFMCW) system diagram.

The radar analyzes the target information by transmitting electromagnetic waves and receiving the echo signal, which is extracted and processed. The radar system consists of a transmitter, a receiver, a mixer, and an antenna. The radar generates the original signal through the transmitter, and the receiver receives the echo signal reflected from the target object. This process is achieved by transmitting and receiving the signal through the antenna (see Figure 1). An mm-wave radar is sensitive to the measurement of the motion of objects in a fixed background, and it is advantageous for achieving signals from human vital signs, such as heartbeat and respiration, so this design uses mm-wave radar for measurement.

### 2.1. Principle of Operation of Doppler Transceiver

The mm-wave radar picks up the information of the target object by receiving the echo signal through the receiver, and the zero inverse Fourier (IF) method is used for the signal extraction method in this design. The zero IF extraction signal is for the echo signal and is directly mixed with the carrier. Figure 1 depicts the zero IF receiver pickup signal method. A mm-wave radar for heartbeat respiration signal detection is for the detection of the heartbeat movement of the chest cavity. The electromagnetic wave is emitted by the antenna and reflects when it reaches the chest cavity, and due to the motion of the target object, i.e., the chest cavity, there is a frequency shift of the return signal. The electromagnetic wave detects the motion of the target object at a speed *v*(*t*), and the frequency of the echo signal will change as [16]:

(1) $$fa(t)=\frac{2v(t)}{c}f$$

where *fa*, *f* is the echo frequency and the transmitted wave frequency in Hz. Equation (2) is defined for a human motion *w*(*t*), and the phase change inof the echo signal is expressed as below [16]:

(2) $$\varphi (t)=\frac{4\pi }{d}w(t)$$

The phase change of the echo signal is modulated by the change in the thoracic motion state. The heartbeat and respiration signals are extracted separately by analyzing the time-varying characteristics of the echo signal frequency and the frequency characteristics of the heartbeat and respiration. The amplitude of the echo signal changed from the amplitude of transmitted electromagnetic waves due to the reflection from the chest cavity and the attenuation in the propagation process. The frequency shift of the echo signal is mainly detected in the method of detecting vital signs. The expression of the emitted electromagnetic wave is expressed as below [16]:

(3) $$A(t)=A\left[\cos(t-\frac{2d}{c})+\varphi_{0}\right]$$

Here, the initial noise of the emitted electromagnetic wave is assumed to be a random fluctuation in phase. The distance between the chest cavity and the antenna is *d*_0_, the equation of motion of the chest cavity is *x*(*t*), and then the distance between the receiver and the target chest cavity is *d*(*t*) = *x*(*t*) + *d*_0_. Due to the distance between the transmitter and receiver of the chest cavity, the time of electromagnetic wave propagation can no longer be neglected. In the analysis of the reflected signal it can be found that the amplitude of the echo signal will be attenuated, while the phase of the echo signal will be affected by the distance between the transmitting antenna and the detected object due to a certain degree of delay. The relative motion, because of the influence of the chest motion on the echo signal, will also modulate the phase of the echo signal. Finally, the echo signal is pre-processed at the receiving antenna, mixed with the receiver’s internal local oscillator signal, the spurious interference is filtered out, and the filter to obtain the baseband signal filters out the carrier wave. After the quadrature demodulation, the receiver’s internal mixing and demodulation processing can restore all the original baseband signal information in the echo signal.

### 2.2. Biological Basis of Vital Signs Monitoring

The method of detecting human vital sign signals by mm-wave radar is analyzed from a physiological point of view. A mm-wave radar can collect and send human vital signs signals to manage the human breathing or heartbeat signal, mainly because the human heartbeat or breathing life activity will cause human chest movement. Through the human chest movement form of the acquisition, the carrier wave transmission in the internal transceiver, through the local oscillation signal mixing and high-frequency filtering processing, can get the baseband signal, that is, to get the human vital signs information. The baseband signal is obtained, i.e., the data of human vital signs are obtained. The human vital signs can be collected in the baseband signal because of the human physiological basis. Through the analysis of the frequency domain, it can be found that the frequency of the human heartbeat and respiration is shallow, and the frequency of adult breathing is from 60 to 90 times per minute, while the frequency of adult breathing is from 15 to 24 times per minute. Table 1 shows the characteristics of an adults’ heartbeat and respiratory activity in a normal state.

Among the main methods of detecting vital signs in medicine, the method used to detect respiratory rate is the observation method or chest palpation. This method is inaccurate because the person tested may find that they are tested for the heartbeat and respiratory signals and may change their breathing status, either intentionally or unintentionally, due to stress. At the same time, for some exceptional cases, such as extensive burns, the use of contact measurement is also highly inconvenient, so in such cases, the use of non-contact mm-wave radar for the human vital signs detection method is preferred. Human respiratory or heartbeat signal extraction has essential significance and application prospects. The human vital signs signal amplitude is enough to be extracted by the millimeter wave radar. This study found that the human heartbeat can make a human chest movement of a maximum displacement of 0.6 mm. The maximum amplitude of the human chest caused by exhalation after the experiment could cause 4 mm to 12 mm amplitude. This displacement is sufficient for the radar transceiver to collect and pick up the signal. The mm-wave radar system can extract Doppler frequencies for such a motion state, pick up, and analyze vital human signals [3].

**Figure 2:**
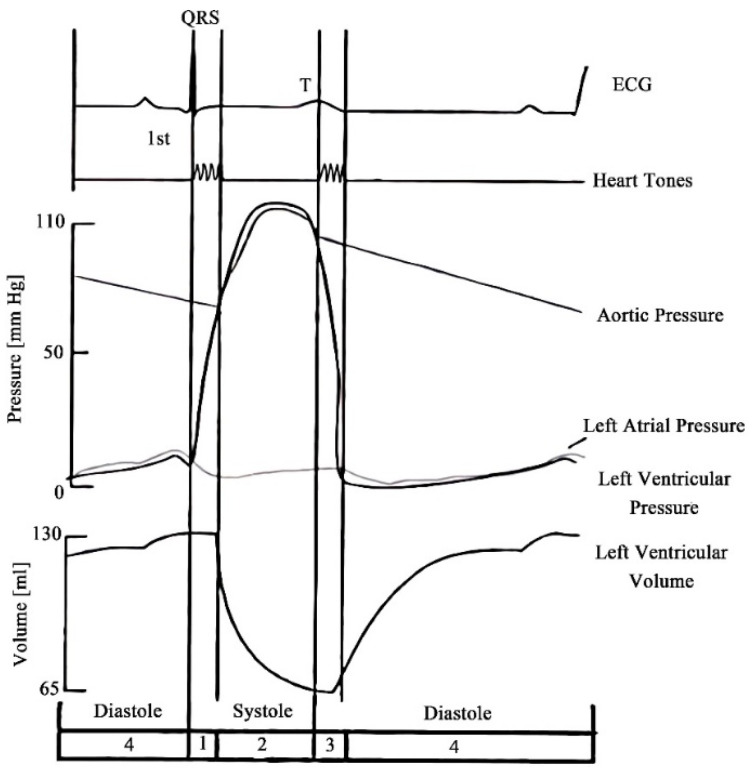
Volume changes during a heartbeat.

The extraction of human vital sign signals using millimeter wave radar is performed by using the phase difference between the transmitted signal and the echo signal on the moving object to obtain the state information of the thoracic motion, thus realizing the volume changes during a heartbeat signals (see Figure 2). However, the signal extraction requires demodulation of the echo signal. For continuous mm-wave bio-radar, there are two ways to demodulate the echo signal: zero secondary IF or choosing a receiver with a super outlier structure. The zero-second IF receiver is used to mix the echo signal with the receiver’s transmit signal so that the echo signal demodulates. The signal is moved to the zero IF, and the frequency result of the IF is extracted in the frequency domain to obtain the frequency value of the heartbeat respiration signal to be measured. In the demodulation of the echo signal using the receiver with the super outlier structure, the result is mixed to the baseband by mixing the echo signal with the local oscillator signal inside the transceiver to achieve the demodulation of the echo signal.

The differential structure is often chosen for the continuous mm-wave bio-radar demodulation process. The receiving antenna collects the echo signal, and a filter performs the first filtering of the echo signal; the amplitude of the original echo signal is increased, and the IF signal can be obtained in the frequency domain. In the demodulation process, if the receiver that selects the IF signal is very suitable, a better measurement result can be obtained when the signal extraction is performed. However, producing a mirror frequency in the demodulation process is easy if the echo signal is relatively similar to the return signal. In such a case, the signal saturation may be caused by the echo signal’s low demodulation speed, and the super outlier structure’s receiver needs to consider the mirror frequency problem in the demodulation process.

In the demodulation process of that frequency-modulated continuous wave (FMCW) radar, zero secondary IF can also be used. In the demodulation process of zero IF, after the receiving antenna of the transceiver receives the echo signal, it is pre-processed by the filter inside the transceiver to filter out the spurious interference. Finally, the pre-processed echo signal is mixed with the local oscillator signal inside the transceiver to obtain the signal in the baseband in the frequency domain analysis. On the one hand, the transceiver with a zero IF structure can avoid the disadvantages mentioned above of the super outlier receiver so that after the zero IF processing of the echo signal, the interference of the mirror frequency is avoided. On the other hand, the transceiver with a zero IF structure can use the same local oscillator signal. In this case, the transceiver can be designed without filters for the mirror frequency, while only one fundamental frequency can be generated, reducing system noise and interference from mixed signals in the entire transceiver system. The zero IF structure transceiver shows a significant advantage in short distance measurement and is beneficial for non-contact human vital signs signal pickup.

### 2.3. Constant False Alarm Rate Monitoring for Biological Signal

In radar signal detection, when the noise or interference power changes, the radar can automatically adjust the detection sensitivity so that the false alarm probability remains constant; this characteristic is called the constant false alarm rate (CFAR). CFAR detection is also known as adaptive threshold detection or automatic detection. Detection probability *P_D_* and false alarm probability *P_FA_* are commonly used in radar systems to measure the performance of target detection. The probability of detection is the probability that the target will be detected. The false alarm probability refers to the probability that a target is judged to be present when it is not present due to noise fluctuations during radar detection using the threshold detection method. Due to the existence of Gaussian noise in the system, if $P_{D}\neq 0$ it can be derived from $P_{FA}\neq 0$ that there will certainly be false alarms, and the greater the system noise, the lower the signal-to-noise ratio, and the more likely it will have false alarms.

Let us first derive the relationship between the false alarm probability, threshold, and noise power. After passing the Gaussian white noise through a narrowband IF filter, a narrowband random process ξ(t) is obtained, which can be expressed as

(4) $$\xi (t)=a_{\xi }(t)\cos[\omega_{c}t+\varphi_{\xi }(t)]\;\;a_{\xi }(t)\geq 0.$$

According to the probability theory and random signal analysis, the envelope $a_{\xi }(t)$ is Rayleigh distributed, and the phase $\varphi_{\xi }(t)$ is uniformly distributed. Let the noise power $\sigma_{w}$ and the expression for the probability density of the Rayleigh distribution be as follows:

(5) $$a_{\xi }(x)=\frac{x}{\sigma_{w}^{2}}\exp(-\frac{x^{2}}{2\sigma_{w}^{2}})\;\;\;x\geq 0.$$

**Figure 3:**
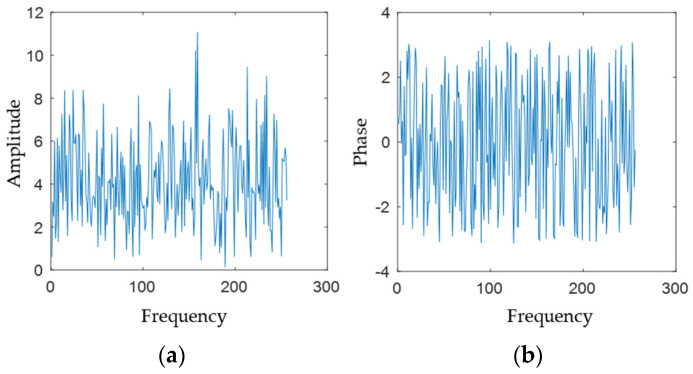
The diagrams of (**a**) amplitude and (**b**) phase of Gaussian white noise obtained by FFT.

**Figure 4:**
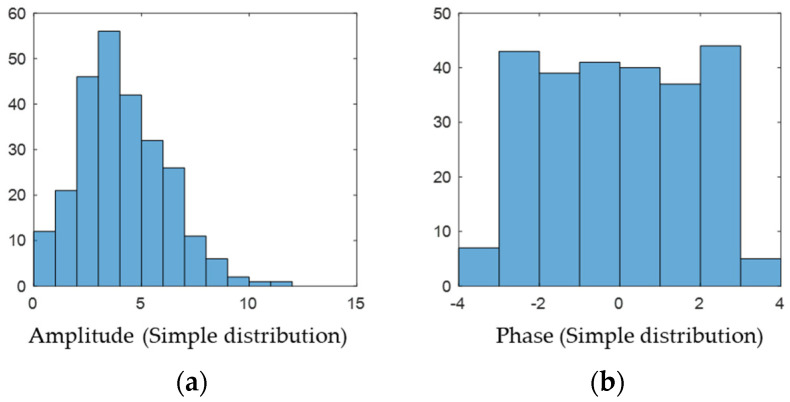
Histogram of the distribution of (**a**) amplitude and (**b**) phase.

The sample distribution is statistically presented, and it can be observed that the amplitude–frequency characteristics are concentrated around 4 (see Figure 3 and Figure 4), with few larger and smaller values, which is consistent with the Rayleigh distribution; the phase frequency characteristics are uniformly distributed between [−π,π].

**Figure 5:**
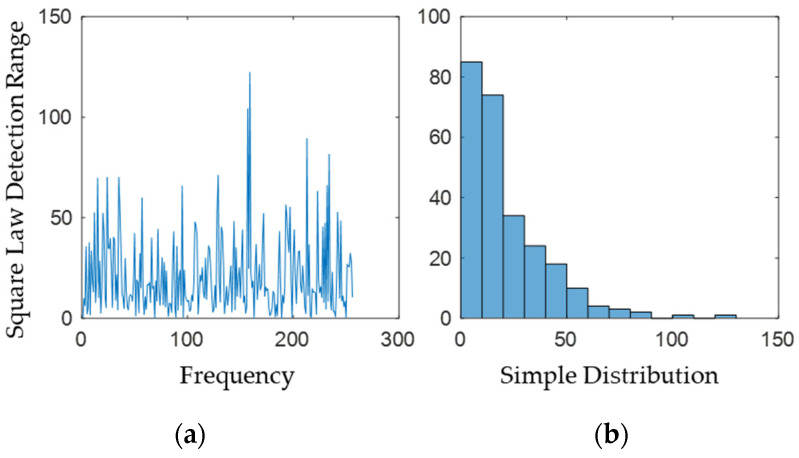
(**a**) Frequency and (**b**) simple distribution after square-law wave detection range.

According to the theory of coherent and incoherent detection of radar signals, the FFT is equivalent to matched filtering of unknown phase signals, and to achieve an optimal detector; the FFT result needs to be modulo the FFT result and then passed through a nonlinear operator consisting of a Bessel function and a natural logarithm $\ln[I_{0}(x)]$, and finally a non-coherent accumulation summation. This nonlinear operator is arithmetically large, unsuitable for hardware implementation, and can be simplified by approximation. Using a level expansion, it can be deduced that $\ln[I_{0}(x)]$ approaches the linear detection operator *x* at low signal-to-noise ratios and the square rate detection operator *x*^2^ at high signal-to-noise ratios. The implementation of the square law operator only requires the sum of squares for the real and imaginary parts, while the implementation of the linear operator also requires the square root, which is not conducive to hardware implementation; and the square law detection is equivalent to getting the power spectrum more conducive to the estimation of the noise power (see Figure 5).

With the addition of the square law test, the probability density distribution changes from Equation (5) of the Rayleigh distribution to an exponential distribution

(6) $$p_{\xi }(x)=\frac{1}{\sigma_{w}^{2}}\exp(-\frac{x}{\sigma_{w}^{2}})\;\;\;x\geq 0.$$

For a given threshold *T*, it is sufficient to calculate that Equation (6) is the integration from *T* to +∞ yield the false alarm probability *P_FA_*:

(7) $$P_{FA}=e^{-T/\sigma_{w}^{2}}.$$

And furthermore,

(8) $$T=-\sigma_{w}^{2}\ln P_{FA}.$$

Therefore, to implement CFAR under the square law detection condition, i.e., *P_FA_* is a constant, and it is only necessary to make the threshold proportional to the noise power. So, the focus is on estimating the noise power, and the main difference between the different CFAR algorithms is the various methods of calculating the noise power.

## 3. Targeted Frequency Detection Algorithm

### 3.1. Thoracic Motion Target Detection

**Figure 6:**
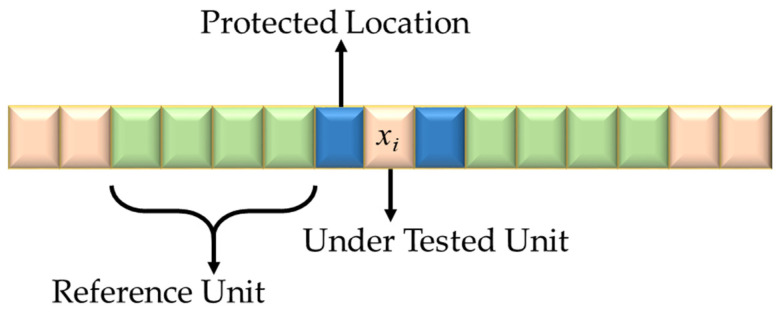
Average police rate projection.

The information is carried by the mm-wave radar, and after the receiving antenna extracts the echo, the movement state of the chest cavity can be obtained and further separation pickup can get the human respiratory heartbeat signal that needs to be detected. In processing the echo signal, the transmitting wave will be affected by the reflection of objects, such as the ground. There will be a lot of clutter interference, but the noise in the reflected wave also needs to be filtered. Because the noise clutter and other interference exists more and will affect the extraction of useful information, there needs to be a design to highlight the valuable signal. In the study of noise and clutter filtering, the method used is to divide the cells near the detection sequence, such as selecting 30 or 40 sequence unit cells, calculating the average value of the clutter in the vicinity of the target to the side of the target, and using this average value to replace the clutter at the predicted target location, which is essentially a maximum likelihood estimation of clutter energy (see Figure 6). In the maximum likelihood estimation method, the estimated clutter energy is used to facilitate the design of the target detection threshold. If the energy of the echo signal can complete the trigger for the target threshold and the detection of the moving target is achieved, then the detection of the thoracic motion is completed. The design of the detection threshold by the method of average police rate is conducive to improving the accuracy and stability of thoracic motion detection.

### 3.2. Moving Target Display Algorithm

**Figure 7:**
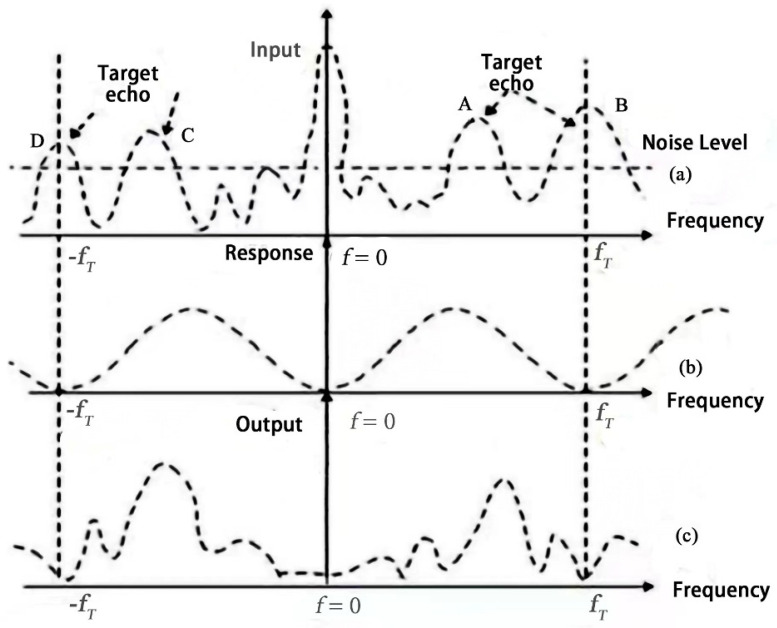
MTI spurious filtering.

In vital sign measurement, mm-waves are used to detect the motion of the chest cavity, but the CW radar propagates to the receiving antenna with many echoes from non-detected targets, such as other animals, plants, and the ground. The energy analysis of the spectrum of discrete sequences in RF signals reveals that the energy at low frequencies is generally low, so the separation of clutter and target frequencies can be achieved using such a method. The echo signal acquired by the receiving antenna is linearly passed through the moving target display filter to filter out the spurious signal at the low frequency and complete the separation of the target signal (see Figure 7). Among the echo signals reflected from the object, the main clutter signals mainly originate from the reflection of the ground, and these echo signals are mainly concentrated at zero frequency. In this design, the frequency detection process is selected for CW radar. The time domain analysis of the signal is very complex, so the FFT method is performed on the radar signal. The signal processing in the frequency domain for the characteristics of the clutter is concentrated in the zero frequency. It is required to filter out the clutter and improve the signal-to-noise ratio. The design of a high-pass filter to complete the filtering of the clutter is conducive to highlighting the target detection signal and further target signal spectrum analysis. It is possible to achieve the heartbeat breathing signal.

### 3.3. Vital Sign Spectrum Extraction Algorithm

The spectral characteristics of heartbeat respiration are more pronounced, with both signals having a lower frequency. Based on current physiological studies, it can be found that under normal conditions, the human heartbeat is essentially between 60 and 100 beats per minute, while the respiratory rate is even lower, at about 18 to 24 beats per minute. It can be used to detect the movement of the human chest cavity. During the movement of the chest cavity, a Doppler shift of the echo signal is produced, which completes the pickup of the heartbeat or respiratory frequency. The chest motion caused by breathing is about 12 mm per time, while the heartbeat only shifts about 0.5 mm. It is easier to achieve the detection of breathing frequency with the mm-wave radar. Due to the small displacement caused by the heartbeat, the detection process of the heartbeat is more easily disturbed by other clutter and not easily separated from the spectrum of the breath, so pre-processing of the target signal is required.

#### 3.3.1. Target Signal Pre-Processing

**Figure 8:**
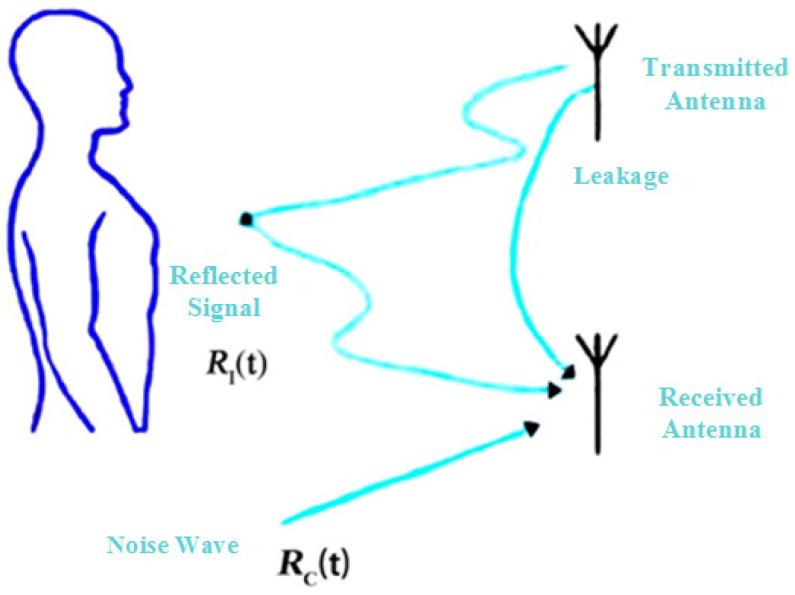
Transmitting antenna leakage.

The above analysis is about the clutter affecting the heartbeat frequency detection when the spectrum of the heartbeat signal needs to be picked up. The echo signal must first be pre-processed to enhance the energy value corresponding to the prominent heartbeat spectrum. The mm-wave radar exposure to the external environment requires consideration of the following three types of clutter interference in addition to the echo signal that produces the Doppler effect after thoracic motion. First is the impact of white noise received by each group of acquired sequences during the real-time measurements performed by the mm-wave bio-radar. The thoracic motion will reflect the transmitted mm-wave radar, but the receiving antenna will then pick up other reflections from the human environment, such as the ground (see Figure 8). The most important part is to consider the concentrated energy distribution in the clutter because the signal reflected from the whole environment will be much more than the signal reflected from the chest cavity alone, compared to the amplitude of the chest movement. The last part of the spectrum is the leakage of the initial carrier signal in the transceiver system directly past the transmitting antenna to be received by the receiving antenna.

#### 3.3.2. Vital Sign Phase Detection Method

The above analysis about the mm-wave bio-radar for the heartbeat detection process shows that, due to the heartbeat process caused by the thoracic displacement, an amount of displacement less than 0.5 mm in the mm-wave radar reflection process caused by the Doppler frequency shift is more difficult to pick up. This design considers using a set of input sequences, the phase difference of the adjacent set of discrete sequences, to carry out the extraction of the spectrum.

**Figure 9:**
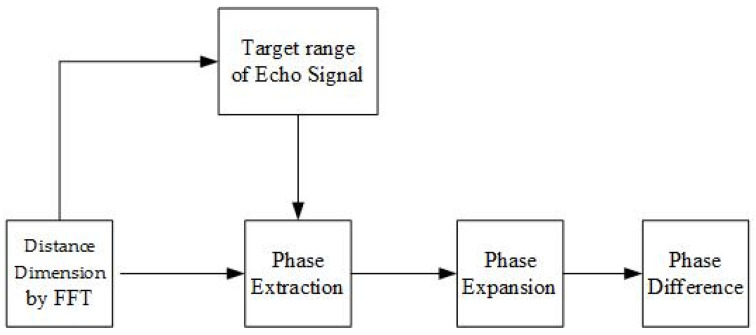
Phase detection flow.

In the process of extracting the heartbeat spectrum using the phase detection method, the focus is on converting the measurement for the thoracic displacement to the phase change of the echo signal, where the measurement of the phase is performed and there is a need to keep the human distance between the transceiver system and the human body constant at all times. When measuring the phase change of a set of input signals, mm-wave radar first needs to find the maximum value of a set of data for the discrete input sequence and measure the corresponding phase value for this maximum value. Finally, the phase difference of the discrete sequence is measured, i.e., the phase difference between two adjacent points in the discrete sequence. If the phase difference between two discrete points is more significant than zero, it means that the phase of the former point is ahead of the phase of the latter point (see Figure 9). In addition, in performing the stability design of the phase difference, this design utilizes phase expansion.

#### 3.3.3. Moving Target Indication (MTI) Filter Designed Algorithm

Contact cardiac and respiratory detection methods, usually using an array of pressure sensors or strain gauges, are applied to the chest, and when the heart beats and the chest heaves, the acquired pressure signal is converted to a sensor conditioning circuit, converted by A/D, and then processed by a digital signal processor. Noncontact FMCW radar is applied for vital signs monitoring. FMCW radar detects moving targets, often only interested in moving targets; at this time, the static material echo information in the test environment becomes useless clutter, bringing a more incredible difficulty to the identification of moving targets. Therefore, one of the necessary conditions for the radar to work properly is to eliminate static clutter. However, in the time domain, signal processing is difficult to distinguish between the target echo and clutter signal. Therefore, radar is used to indicate (or identify) moving targets, so as to suppress clutter in the frequency domain. MTI is a method to improve the identification of the motion target signal to be measured by suppressing various forms of clutter interference in the signal through the filter principle.

The design of MTI, essentially a high-pass filter design, can be implemented using algorithms, such as IIR, FIR digital filters, wavelet algorithm, and empirical modal decomposition. Wavelet transform and practical modal decomposition methods are also essentially filtering processes and decompose the signal with harmonics of different scales one by one, generating local frequency domain features of different characteristic scales to the target to be measured and adapting according to signal characteristics to adapt to the identification and analysis of the target in order to achieve optimal filtering. However, their complexity and resource occupation are much more significant than IIR and FIR digital filters. Due to the relatively complex recursive adaptive filtering structure, the wavelet algorithm and empirical modal decomposition algorithms are less real-time than IIR and FIR digital filters.

MTI filters generally require as little complexity and resource consumption as possible, so a delayed pair canceller is commonly used to implement MTI in radar systems. It is also essentially a low-order FIR filter, which approximates optimal FIR filtering for random velocity targets, but has poor selectivity for low-velocity targets. Compared to FIR, IIR can achieve good selectivity with few orders, low resource consumption, high efficiency, and low design complexity. However, there are many disadvantages: the excellent selectivity of IIR is traded for a nonlinear phase, IIR has a recursive structure and rounding errors in fixed points may cause the poles to deviate outside the unit circle, resulting in an unstable system, and the values inside the recursive structure may be huge, requiring high dynamic range.

However, the proposed work applies FMCW radar with the IIR filter acting on slow time dimensional data. Although the phase of the IIR is nonlinear, it only affects the initial phase, and the phase difference is constant, so the nonlinear phase does not affect the distance and velocity measurements. In addition, the rounding error problem of the low-order IIR is not severe, and the instability can be avoided by paying attention to quantization issues during design. The dynamic range problem can be improved by changing the implementation structure and can be solved by adjusting the number of fixed points. Finally, this radar system needs to measure low-velocity targets, which requires high selectivity, and our design provides excellent selectivity. In summary, the MTI filter system based on IIR filtering is selected for this design.

The design of MTI, essentially a high-pass filter, can be implemented with IIR and FIR digital filters. MTI filters generally require as little complexity and resource usage as possible, so delayed pair eliminators are commonly used in radar systems to implement MTI. It is also essentially a low-order FIR filter, which approximates optimal FIR filtering for random velocity targets, but is less selective for low-velocity targets. Compared to FIR, IIR can achieve good selectivity with few orders, with less resource consumption, high efficiency, and less design complexity. However, there are many drawbacks: the excellent selectivity of IIR is traded for a nonlinear phase; IIR has a recursive structure, and rounding errors in fixed points may cause the poles to deviate outside the unit circle, leading to an unstable system and the values inside the recursive structure may be very large, requiring high dynamic range.

For FMCW radar, the IIR filter acts on the slow time dimensional data. Although the phase of the IIR is nonlinear, it only affects the initial phase, and the phase difference is constant, so the nonlinear phase does not affect the distance and velocity measurements. The rounding error problem of the low-order IIR is not severe, and the instability can be avoided by paying attention to quantization issues during design. The dynamic range problem can be improved by changing the implementation structure and can be solved by adjusting the number of fixed points. This radar system needs to measure low-speed targets with high selectivity requirements. The high-pass filter metrics should be determined based on the actual clutter’s power spectrum and the moving target’s frequency range, with the clutter frequency determining the stopband and the target frequency determining the passband. The assumed metrics are passband cutoff frequency $\omega_{p}$ = 0.02π, maximum passband attenuation $\alpha_{p}$ = −3 dB, stopband cutoff frequency $\omega_{s}$ = 0.002π, stopband maximum attenuation $\alpha_{s}$ = −20 dB, and $\Omega_{s}$ and $\Omega_{p}$ represented normalized passband and stopband frequency.

Then the order *N*

(9) $$N=-\frac{\mathrm{lg}(\sqrt{\frac{10^{\alpha_{p}/10}-1}{10^{\alpha_{s}/10}-1}})}{\mathrm{lg}(\Omega_{s}/\Omega_{p})}=0.9988\approx 1.$$

The transfer function of the first-order analog high-pass filter is

(10) $$H(s)=\frac{s}{s+\Omega_{p}}=\frac{s}{s+\omega_{p}/T}\;\;\;\;\;∵s=jw.$$

Bringing in the bilinear transformation Equation

(11) $$s=\frac{2}{T}\frac{1-z^{-1}}{1+z^{-1}}.$$

Obtain *H*(*z*) and the difference Equation (*T* is the sampling period of Doppler radar).

(12) $$H(z)=\frac{\frac{2}{T}\frac{1-z^{-1}}{1+z^{-1}}}{\frac{2}{T}\frac{1-z^{-1}}{1+z^{-1}}+\omega_{p}/T}=\frac{1-z^{-1}}{\left(\frac{\omega_{p}}{2}+1\right)+\left(\frac{\omega_{p}}{2}-1\right)z^{-1}}=\frac{Y(Z)}{X(Z)}$$

(13) $$y(n)=\frac{1}{\frac{\omega_{p}}{2}+1}\left[x(n)-x(n-1)\right]-\frac{\frac{\omega_{p}}{2}-1}{\frac{\omega_{p}}{2}+1}y(n-1).$$

Bringing in $\omega_{p}$ = 0.02π rad gives

(14) y(n)=0.9696[x(n)−x(n−1)]+0.9391y(n−1).

To facilitate hardware implementation and reduce resource consumption, this is quantified to 5 bits.

(15) $$y(n)=\frac{31}{32}\left[x(n)-x(n-1)\right]+\frac{30}{32}y(n-1)$$

**Figure 10:**
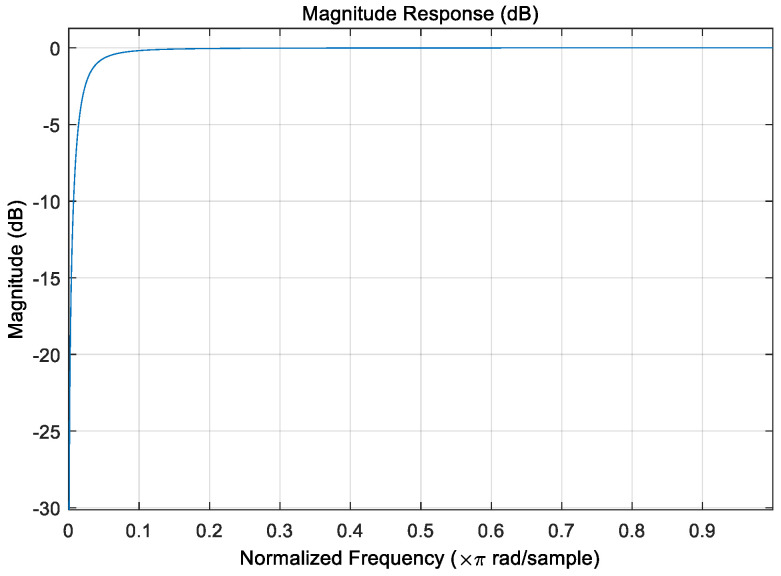
Amplitude and frequency response of the designed filter.

Importing the filter parameters into MATLAB, the amplitude–frequency response is obtained in Figure 10.

After determining the coefficients, a choice of implementation structure needs to be made; for this design, a transposed direct type II structure was chosen for the proposed study. The designed IIR filter cannot be used directly for MTI yet because MTI is the slow time dimension I/Q data processing. Assuming that the number of samples per chirp in the fast time dimension is *N*, the filtering interval of MTI is N, and 2N sets of filters have to be completed, i.e., MTI is equivalent to two N-channel IIR filters. To implement an N-channel filter, each single delay unit in the filter must be replaced with an N-delay unit. During the step-by-step operations in the FFT, data overflow is prone. As mentioned, the data may be scaled up by a factor of 28. The most straightforward solution is to scale the input data down by a direct factor of 28, but this has a significant impact on accuracy and is equivalent to 16 bits of fixed points being directly wasted by half. This design uses an alternative overflow control method. The essential operation of the FFT butterfly cell can be expressed as

(16) $$\begin{array}{l} a^{\prime }=a+b\\ b^{\prime }=(a-b)W_{N}^{p} \end{array}\;\;\;\;\;\;\;∵W_{N}^{P}=e^{-j\frac{2\pi }{N}}$$

where *N*: the operator of DFT, $a^{\prime },b^{\prime },a,b$ are complex numbers and $a^{\prime },b^{\prime }$ are posterior level data, and since the rotation factor is constant modulo 1, it follows that

(17) $$\begin{array}{l} \left|a^{\prime }\right|\leq \left|a\right|+\left|b\right|\\ \left|b^{\prime }\right|\leq \left|a\right|+\left|b\right| \end{array}$$

(18) $$\max\left(\left|a^{\prime }\right|,\left|b^{\prime }\right|\right)\leq \max(2\left|a\right|,2\left|b\right|).$$

Therefore, data growth at each level must be less than or equal to two times. Overflow can be avoided by simply dividing the output of each level of butterfly operation by two or by performing a sign bit expansion at each level. In order to reduce resource consumption, dividing by two at each level is used to avoid overflow in this design. Compared to directly shrinking the input data, this approach, while accumulating the exact multiple of shrinkage, will shrink the noise at each level and end up with very little accumulated noise. Furthermore, note that although multiplying by the rotation factor does not change the modulus of the complex number, it will cause the real and imaginary parts of the data to fluctuate up to $\sqrt{2}$ times the original, so also ensure that neither the real nor imaginary parts of the input data at the first level exceed the maximum $1/\sqrt{2}$.

## 4. Results and Discussion

### 4.1. Validation Environment

The design first requires a joint simulation using Quartus and Modelsim to verify the correctness of the threshold detection module and the moving target pickup module in the digital hardware system, as well as to verify the hardware resource occupation of each module and the implementation of the FFT frequency extraction algorithm. Finally, the algorithm function’s verification and the system’s hardware resource occupation are completed, after the entire system is assembled. The design needs to complete the data extraction verification for the original vital signs signal. Here, the real human vital signs verification signal provided on the official website of TI is used to simulate and verify the FFT frequency pickup hardware algorithm within MATLAB, and the original signal shared by TI about the vital signs experiment is used to complete the simulation verification for the frequency pickup algorithm. The FPGA is used for the final functional verification and timing analysis. While the actual vital signals are extracted and verified, SignalTap is used to display the time results, and a sports bracelet is used to detect the heartbeat and respiration signals in real time to compare the validity and reasonableness of the verification results and compare the error rate.

### 4.2. Results and Analysis of the Detection Module

#### 4.2.1. Threshold Detection

A threshold detection module simulation is performed, where the test signal is mixed with the white noise of high energy while adding sinusoidal signals, each set of which has a different frequency value and energy points.

**Figure 11:**
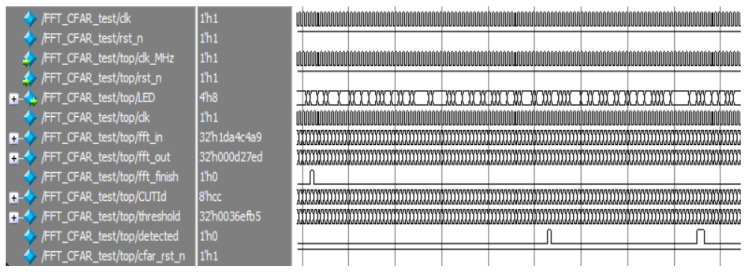
Threshold detection Modelsim results.

**Figure 12:**
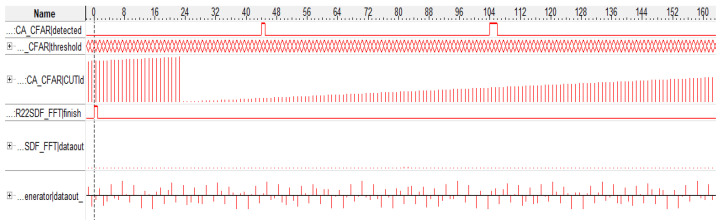
Threshold detection signal-tap results.

As can be seen from the two comparison results, the threshold detection module was simulated and verified in both Modelsim and signal-tap, setting the same noise threshold and performing threshold triggering experiments on the signal in a white noise environment (see Figure 11 and Figure 12). It can be seen that both experimental results show that there was a total of three successful triggerings of the detection threshold, with a sum of two frequency values detected.

**Figure 13:**
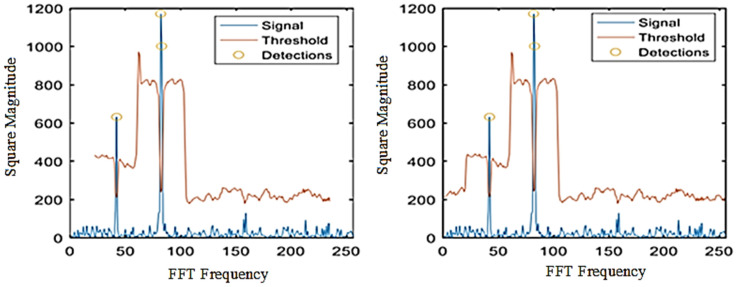
Comparison of the effect of threshold detection.

As shown in Figure 13, the left side shows the threshold detection experiment using the library function provided in MATLAB, and the comparison with the algorithm on the right side shows that the output threshold function is the same around the frequency detection. In the original signal, the detection threshold is triggered at all three frequencies to complete the pickup for the spectral values, and the results are the same.

**Figure 14:**
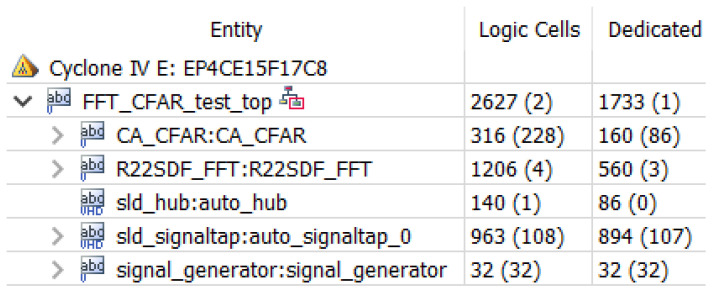
Threshold detection resource occupancy.

The hardware resources occupied by the threshold detection module are shown in Figure 14 above, where CFAR, i.e., average threshold detection, is the threshold detection module used in this design. Using average CFAR consumes fewer resources, and the module occupies only 316 arithmetic logic units.

#### 4.2.2. Moving Target Detection

To carry out the software detection process of the moving target detection module, the input signal used is still a sine wave, and here a linear FM signal method is used to modulate the carrier frequency, because such signals have better resolution for moving targets. The detection of multiple targets can be achieved at increased distances, and the signals selected have different energies.

**Figure 15:**
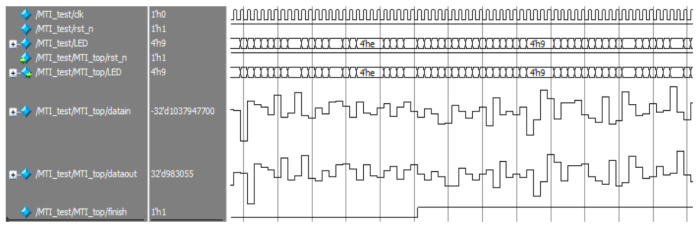
Moving target detection before filtering.

**Figure 16:**
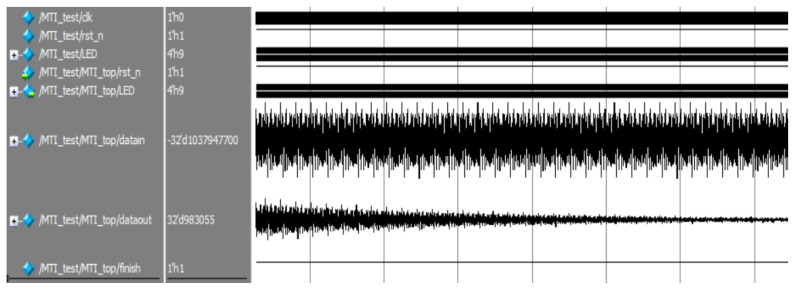
Moving target detection after filtering.

Figure 15 depicted the effect of the input signal after filtering by moving target detection, where there are two signals in each group, the upper one is the input signal of the module, and the latter is the output signal. Figure 16 shows the image of the initial part of the signal after amplification in the previous figure, and it can be seen that the signal gradually stabilizes after the moving target detection module.

**Figure 17:**
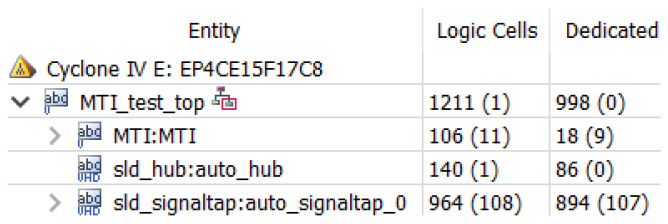
Moving target detection resource occupancy.

Figure 17 represented the hardware resource occupation for moving target detection, where the MTI module, which is the designed moving target indication module, occupies about 100 arithmetic units.

#### 4.2.3. Overall Module

During the overall system simulation, the moving target detection, signal pickup algorithm, and threshold detection modules need to be assembled in white noise with high energy values, with a sinusoidal signal added to each set of test signals and with frequency modulation of the carrier signal, each set of added test signals having a different energy.

**Figure 18:**
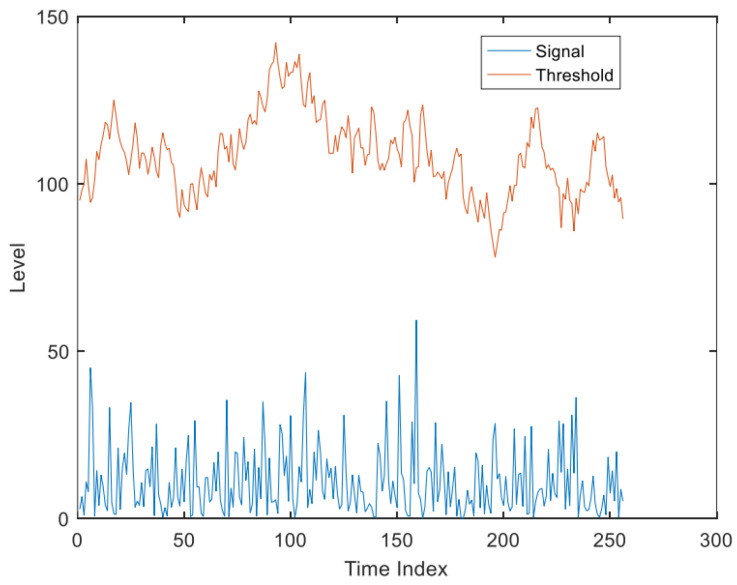
Threshold function output after moving target detection.

In Figure 18, the output signal after moving target detection is shown below, and the threshold function signal in a white noise environment is shown above. After the moving target detection filtering, the stationary target is filtered out and not detected.

**Figure 19:**
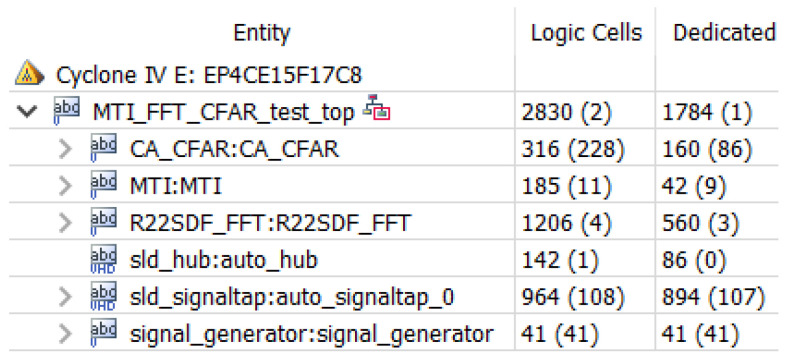
Occupancy of FPGA resources for moving target detection.

**Figure 20:**
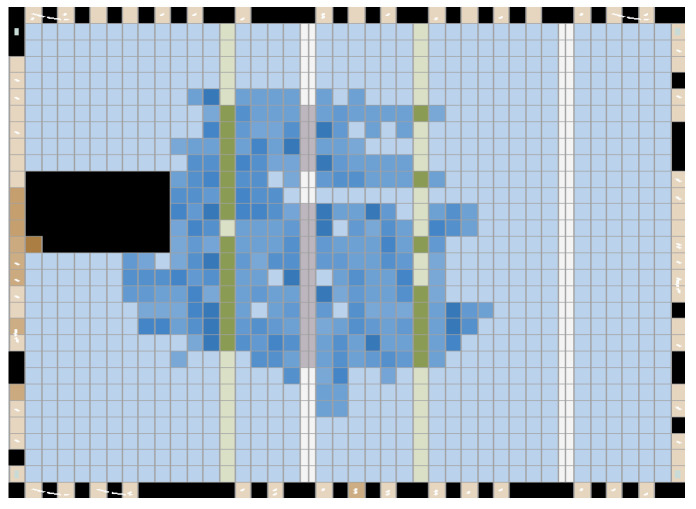
Hardware resources planner of FPGA.

After assembling the three modules, the hardware computing resources occupied by the three modules and the final layout wiring results hardware resources of FPGA are shown in Figure 19 and Figure 20, respectively, and it can be seen that the final hardware resources occupied are more than 1700 logic units.

### 4.3. Signal Data Detection

**Figure 21:**
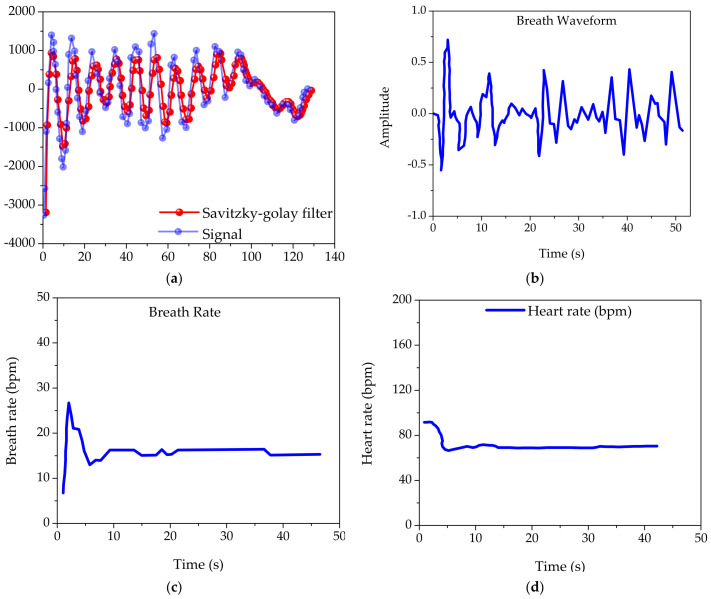
(**a**) Savitzky-Golay filter and signal waveform; (**b**) breath waveform signal; (**c**) breath rate signal waveform; and (**d**) heart rate curve.

The experiments are based on the official vital signs experimental sample provided by TI, and the original echo signal supplied by it is selected for filtering, and the results are shown in Figure 21a. The actual echo signal of the vital sign detection provided by TI and the result after processing is shown in Figure 21a. The echo signal in the plotted graph is the blue line, and it can be seen that the signal represented by the blue line has more clutter interference, and the waveform does not have typical characteristics. The original signal is filtered to remove the relevant clutter, retaining the information originally containing the vital signs signal, while making the signal waveform more regular, the waveform smoother, and the sampling points for fitting are left on the output red signal waveform.

The spectrum extraction process is performed on the fitted output signal represented by the above output red line, and here the spectrum of the useful heartbeat respiration signal can be extracted after an effective estimation of the range of the spectrum based on the analysis of the human vital sign characteristics. Figure 21b shows the time domain results of the original respiration signal waveform. In the section on the analysis of vital characteristics, an adult’s normal vital activity state is indicated, and the filter selection is based on the set spectrum range.

Figure 21c,d show the breath rate and heart rate signal waveforms. Analysis of the final results reveals that in the steady state, the frequency of the heartbeat is about 83 breaths per minute, while the frequency of breathing is about 16 beats per minute; both results are within a reasonable prediction range and are the same as the official TI comparison results, verifying the feasibility of the algorithm.

(19) $$\mathrm{Relative}\;\mathrm{Error}\;(\%)=\frac{\mathrm{Measured}\;\mathrm{Value}\;-\;\mathrm{Reference}\;\mathrm{Value}}{\mathrm{Measured}\;\mathrm{Value}}$$

To evaluate the recognition accuracy of real-time vital sign detection algorithms, the heart rate is considered an example; biometric radar and motion bracelet or pulse sensors were used to synchronise human heartbeat information in the experiment. The sports bracelet makes it easier to extract pulse rate information. As a reference signal, the vital signs radar is still located at the human target ≤ 0.5 m, according to the radar radiation beam on the human heart rate and respiration detection.

The results shown in Table 2 are the results of the measurement and comparison of vital signs under 15 different exercise states, respectively. The relative error of the radar measurement and the bracelet measurement results are measured after exercise and rest for the heart rate measurement. The relative error of the radar measurement is 0–6.3%, and most of the measurement relative error is less than 5%. The relative error for respiration rate measurement is 0–9.5%. Table 3 highlights recent publications of quantitative Doppler radar work on periodic displacement measurement. With the findings in this research, measurement accuracy in terms of displacement range and average error range are compared.

## 5. Conclusions

The proposed design is well accomplished for mm-wave radar-based non-contact vital sign detection in daily life activity. It is mainly oriented toward the analysis and research based on a frequency extraction algorithm. The overall design is finalized with the algorithm of moving target indication and threshold trigger module. The zero IF receiver is selected for picking up the echo signal concerning the frequency characteristics of the extracted thoracic motion and the displacement size. The FFT computation is chosen as a mixed-base method for computing, which integrates the requirements of real-time detection as well as the utilization of hardware resources. The computational structure is preferred to be carried out in parallel structure, which significantly improves the computational speed, and the final verification finds that 1784 computational logic units are occupied, and the hardware resources are highly utilized. The real-time detection of vital sign signals and display on the signal-tap platform were successfully achieved through FPGA verification. The heartbeat and respiration were detected using the sports bracelet, and the relative errors of heartbeat detection were 0–6.3%, respiration detection was 0–9.5%, and the relative errors of heartbeat detection were overwhelmingly less than 5%. The respiration was prone to more significant errors because of the small base fluctuations; but most of the detection is more accurate, verifying the feasibility of the non-contact vital sign detection method.

## Figures and Tables

**Table 1:** Characteristics of respiratory and heartbeat activity.

Life Activity	Frequency (Hz)	Thoracic Displacement (mm)
Heartbeat	0.6–2	0.15–0.5
Respiration	0.1–0.5	0.01–0.15

**Table 2:** The mm-wave radar detection vs. sports bracelet detection statistics.

	Heart Rate (Beats per Minute)			Respiratory Rate (Breaths per Minute)		
S. No.	MM-Wave Radar Measurement Results	Sports Bracelets	Relative Error	MM-Wave Radar Measurement Results	Sports Bracelets	Relative Error
1	69	71	2.8%	14	14	0%
2	69	72	4.2%	16	15	6.7%
3	72	75	4%	16	17	5.9%
4	76	74	2.7%	19	21	9.5%
5	85	80	6.3%	19	20	5.0%
6	66	70	5.7%	21	23	8.7%
7	71	74	4.1%	22	22	0%
8	80	80	0%	24	24	0%
9	97	101	4.0%	17	18	5.6%
10	111	106	4.7%	21	20	5.0%
11	70	71	1.4%	22	23	4.3%
12	81	85	4.7%	24	23	4.3%
13	92	89	3.4%	18	19	5.3%
14	105	101	4.0%	27	26	3.8%
15	109	112	2.7%	30	29	3.4%

**Table 3:** Comparison of displacement estimation results with the reported literature.

References	Frequency (GHz)	Method	Sampling Frequency	Target Periodic Motion Frequency (Hz)	Displacement Estimation Range (mm)	Average Error Range in Displacement Estimated (%)
[26]	40	Complex signal demodulation (center estimation, circle fitting method)	N/A	0.33	2	2.3–2.8
[27]	5.46	Complex signal demodulation	N/A	0.6	0.3–1	0.6–11.7
[28]	2.4	Center estimation w/IC	N/A	1.0	10–40	2–7.5
[29]	2.4	Center estimation w/IC + RC (DC coupled)	1 kHz	0.3	10–40	0.14–0.27
[30]	2.4	Center estimation w/IC + RC (AC coupled)	N/A	0.3	4–12	0.01–2.08
[31]	2.4	Center estimation w/RC (DC coupled)	1 kHz	1.0	0.1–1	0. 13–3.88
[32]	2.4	Center estimation w/IC + RC (DC coupled)	N/A	0.2	4–12	0.01–2.63
[33]	77	Complex signal demodulation (FFT, machine learning)	5 Hz	2	0–5	3.85
[34]	8.7	Complex signal demodulation (FFT, MTI, window)	5.5 MHz	N/A	0.5–2.5	5
[35]	6.8	Complex signal demodulation (FFT, correlation method)	110 Hz	0.5	N/A	1.3 (heart rate)/1.2 (respiratory rate)
**This work**	**24**	**Complex signal demodulation (FFT, MTI, CFAR, window)**	**65 MHz**	**0.3**	**100–500**	**0–6.3** **(heart rate)/0–9.5 (respiratory rate)**

## Data Availability

Not applicable.
